# Unexpected breast cancer mimicking benign lesions on ultrasound-guided vacuum-assisted excision biopsy: A retrospective cross-sectional study over a 20-year period

**DOI:** 10.3389/fonc.2023.1108689

**Published:** 2023-02-01

**Authors:** Wu Zhou, Honghao Luo, Haina Zhao, Yulan Peng

**Affiliations:** Department of Ultrasound, West China Hospital, Sichuan University, Chengdu, Sichuan, China

**Keywords:** unexpected breast cancer, vacuum-assisted excision biopsy, clinicopathological feature, late diagnosis, sonographic feature

## Abstract

**Objectives:**

This study investigated the occurrence rate of unexpected breast cancer (UEBC) mimicking benign lesions [Breast Imaging Reporting and Data System (BI-RADS) category 3 or 4a] using ultrasound-guided vacuum-assisted excision biopsy (US-VAEB), and explored the factors responsible for late diagnosis of T2 stage UEBC.

**Materials and methods:**

We collected clinicopathologic data and preoperative US imaging features within 3 months before US-VAEB of patients who were diagnosed with UEBC from January 2002 to September 2022. The UEBC were divided into T1 and T2 stageUEBC. The US imaging features as well as clinical and pathological information of T1 and T2 stage UEBC were compared to explore the factors responsible for late diagnosis of T2 stage UEBC.

**Results:**

Breast cancer was diagnosed in 91 of 19 306 patients who underwent US-VAEB. We excluded eight patients with breast cancer assigned to BI-RADS 4b category by preoperative US, and two for whom US imaging records were unavailable. Finally, we enrolled 81 patients. The occurrence rate of UEBC after US-VAEB was 0.42%(81/19296). Of the 81 cases of UEBC, 22 were at T2 stage. The ratio of T2 stage UEBC was 27.2%. The differences in risk factor of breast cancer and routine breast US screening between T1 and T2 stage UEBC were significant[96.6% (57/59) vs 81.8% (18/22), 44.1% (26/59) vs 13.6% (3/22), respectively, *P*<0.05).

**Conclusion:**

UEBC was rarely detected by US-VAEB. Most cases of T2 stage UEBC were diagnosed late because of the absence of routine US screening and risk factors for breast cancer. Stricter clinical management regulations for breast lesions and performing regular US screening may be helpful to reduce T2 stage UEBC.

## 1 Introduction

Breast cancer is one of the most commonly diagnosed malignant tumors and its incidence is increasing annually. In 2020, female breast cancer surpassed lung cancer, with an estimated 2.3 million new cases (1). Breast cancer is a highly heterogeneous disease (2). Early detection and individual treatment remain key factors to improving therapeutic efficacy as well as prognosis (3). The risk of malignancy of any breast abnormality is evaluated by different diagnostic imaging modalities, such as ultrasonography, mammography and magnetic resonance imaging (MRI) (4). In China, mammography and especially ultrasonography are the primary breast cancer screening methods. MRI is usually performed as a supplemental diagnostic procedure when mammography in conjunction ultrasound fails to achieve diagnosis. The long scan time and high costs have limited widespread use of MRI in breast cancer detection and diagnosis (5). The sensitivity of these imaging methods is limited for differentiation of malignant from benign lesions, especially in dense breast parenchyma, with an average diagnostic efficiency of around 70% (6, 7), which means that some breast cancers are missed. Of these missed breast cancers, some are recommended to be followed up, the others to be treated by minimally invasive surgery, such as ultrasound-guided vacuum-assisted excision biopsy(US-VAEB). For those breast cancers excised *via* US-VAEB, although they are not identified preoperatively by imaging modalities because of their benign appearance, they eventually receive a pathological diagnosis *via* US-VAEB. Viewed in this light, US-VAEB plays an important role in early diagnosis of breast cancer, which yields benefits for patients. US-VAEB has several advantages: obtaining enough samples for reliable histological diagnosis; ability to completely remove breast benign lesions; and performance under real-time US guidance. US-VAEB has become one of the most popular minimally invasive surgical techniques for diagnosis and treatment of breast abnormalities, and one of the most important procedures for early detection and diagnosis of breast disease (8). To date, diagnosis of unexpected breast cancer (UEBC) by US-VAEB has been rarely reported in the literature. This study investigated the occurrence rate of UEBC mimicking benign lesions [Breast Imaging Reporting and Data System (BI-RADS) category 3 or 4a] using US-VAEB, as well as the proportion of T2 stage UEBC, to explore the factors responsible for late diagnosis of T2 stage UEBC

## 2 Materials and methods

### 2.1 Patients and lesions

The Ethics Committee of West China Hospital approved this retrospective study.

This study was conducted between September and October 2022. We retrospectively reviewed the pathological results of all patients who underwent US-VAEB in West China Hospital, Sichuan University between January 2002 and September 2022. We selected patients diagnosed with invasive breast cancer. We excluded patients whose clinical or US imaging data were unavailable or who had BI-RADS 4b or greater breast cancer preoperatively. Subsequently, we enrolled patients with malignant lesions classified as BI-RADS 3 or 4a by preoperative US, which were defined as UEBC.

### 2.2 US imaging

US imaging was performed using a Philips IU22 (Philips Medical Solutions; Mountain View, CA, USA) with a 5-12 MHz linear transducer and a Logiq E9 (GE Healthcare, Milwaukee, WI, USA) with a 5-15MHz linear transducer, supplemented with a convex array probe (1-5 MHz), to penetrate larger masses in dense breast tissue. The bilateral whole-breast US scanning technique was standardized to include lower axillary areas and breast parenchyma. All breast lesions were imaged in two orthogonal planes, covering radial and antiradial or transverse and longitudinal planes. We recorded the clockface location and distance from the nipple of all breast masses. Margin, shape, posterior acoustic pattern, tumor size, orientation, echogenicity, calcification, invasion, blood flow grade, and BI-RADS category were also documented. We evaluated the intratumoral blood supply using the Adler semiquantitative analysis of blood flow grading. Blood flow was graded as follows: grade 0: no blood grow; grade 1: small amounts of flow(one or two punctate or short rod-like color flow signal); grade 2: medium amounts of flow(three or four punctate color flow signals or a longer blood vessel which may be half of the mass dimension long); grade 3: rich flow(more than four punctate color flow signals or two longer blood vessels) (9). We performed BI-RADS category for each breast lesion according to ACR Breast Imaging Reporting and Data System (10). For US-VAEB, we recorded each excised lesion in detail, including location, distance from the nipple, and ordinal number of surgical excision. We saved preoperative, intraoperative and postoperative US images. All ultrasound documents were acquired from the Picture Archiving and Communication System of the Department of Ultrasound.

### 2.3 US image interpretation

All patients had preoperative US examinations within 3 months and images were accessible. If a patient had more than one preoperative US examination, the one closest to the time of diagnosis of breast cancer was used for the analysis. We identified the US images of UEBC according to the location mentioned in the pathological report, and invited two radiologists (X.Y.P. and W.Z. with 5 and 8 years of US imaging experience, respectively) to interpret the images of UEBC. They were blinded to the clinicopathological results and original US reports, as well as the exact aims and procedures of our study. They described the sonographic features and made final assessment for each of the UEBCs according to the US-BI-RADS lexicon. The interobserver agreement for lesion descriptors and BI-RADS category was calculated.

### 2.4 Retrospective collection of clinical and pathological data

The clinical and pathological data consisted of patient age at diagnosis, body mass index, history of smoking and alcohol consumption, family history of breast cancer, history of benign breast lesion biopsy, age at menarche, history of abortion, age at first childbearing, history of hormone replacement therapy, history of diabetes, history of high blood pressure, breast parenchyma type, number of risk factors of breast cancer, number of excised lesions in cancerous breast, history of routine breast screening, clinical T stage, histological grade, and immunohistochemical subtype. The pathological pattern was based on *The 2019 World Health Organization classification of tumors of the breast (* 11). All clinical and pathological data were obtained from the Hospital Information System.

### 2.5 Breast lesion excision by US-VAEB

US-VAEB was performed with the patients in the supine position using the 8-gauge biopsy needle of Mammotome^®^ biopsy system (SCM23; Devicor Medical Products, Cincinnati, OH, USA). All breast masses were located on the body surface with US guidance and marked with small dots. The incision position took into consideration the efficiency and convenience of operating the biopsy device, as well as the patient’s desire for a good cosmetic outcome. The skin around the incision placement was disinfected. The high-frequency linear array transducer was used to provide real-time US guidance. The transducer was covered with a sterile glove. Local anesthetic comprising lidocaine and epinephrine (200 000:1, single dose ≤400 mg) was injected into the subcutaneous fat layer above the target lesion as well as the retromammary space underneath the target lesion. The 8-gague Mammotome was positioned under US guidance to ensure the aperture of the needle was just beneath the lesion. The extent of the resection was such that there was a negative surgical margin and no remaining tumor was identified by US, which reduced the risk of tumor recurrence. Benign-looking lesions were resected first, followed by suspicious malignant lesions. If the lesions were distributed in both breasts, the biopsy needle used to excise lesions on one side was not reused on the contralateral side, in an attempt to avoid implantation metastasis. For lesions located close to the pectoralis major or skin, a suitable amount of lidocaine and epinephrine was injected around these lesions so as to create enough space for the hand-held Mammotome device, to avoid damage to skin or chest wall. After the Mammotome needle was removed from the incision, the operative field was rescanned carefully with a high-frequency probe to ensure complete excision of the target lesion. The operating procedure was terminated once no residual tumor was found on US. The hematocele was squeezed out of the breast, and the incision was compressed by elastic bandage to avoid active bleeding for at least 24 h. All excised specimens were stored in formalin, and transferred to the Department of Pathology for histopathological diagnosis.

### 2.6 Statistical analysis

Statistical analysis was performed using SPSS version 25.0 (IBM, Armonk, NY, USA). The Shapiro–Wilk test was applied to verify whether data were distributed normally. Normally distributed data were represented by mean ± standard deviation, and non-normally distributed data by median and interquartile range. The continuous variables were analyzed using the independent samples *t* test. The categorical variables were analyzed by χ^2^ test, Fisher’s exact test or Mann–Whitney *U* test. The interobserver agreement was evaluated with Cohen’s κ test. The κ value was interpreted as suggested by Landis and Koch: poor agreement: ≤0.2; fair agreement, 0.21–0.40; moderate agreement, 0.41–0.60; substantial agreement, 0.61–0.80; and perfect agreement, 0.81–1.0. Two-tailed *P*<0.05 was considered statistically significant.

## 3 Results

### 3.1 Demographic and clinicopathological results

**Figure 1:**
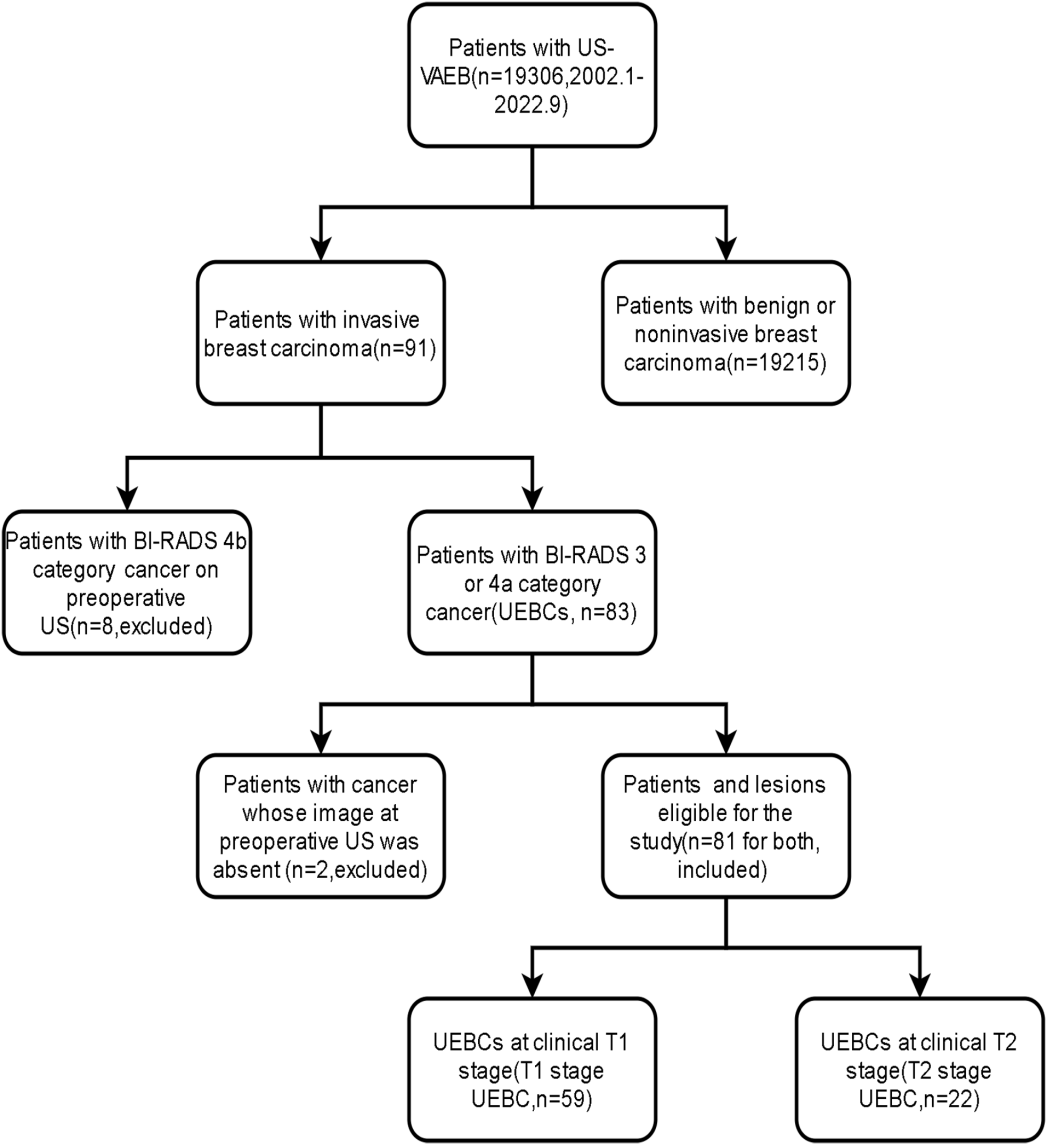
Flow chart of identifying the study population.

Between January 2002 and September 2022, 19 306 patients underwent US-VAEB, and 91 were diagnosed with invasive breast carcinoma. Of the 91 invasive breast carcinomas, 8 were assigned to BI-RADS 4b category on preoperative US examination, and 2 had no corresponding US images on preoperative US examination. These 10 patients were excluded from our study. Finally, 81 cases with 81 lesions were enrolled (**Figure 1**). The occurrence rate of UEBC was 0.42% (81/19296). Of the 81 UEBC, 22 were at T2 stage, 59 at T1 stage. The ratio of T2 stage UEBC was 27.2% (22/81). In T1 stage UEBC, 96.6%(57/59) had risk factor of breast cancer, 76.3%(45/59) presented with dense breast parenchyma. 44.1%(26/59) performed routine ultrasound screening for breast cancer. The differences in breast parenchyma, risk factors for breast cancer, and routine breast screening US were significant (**Table 1**, *P*<0.05). The pathological results showed that 93.8% (76/81) of cases were invasive ductal carcinoma, 17.3% (14/81) were poorly differentiated (histological grade III) breast cancer, 30.0% (17/81) were triple-negative breast cancer, and 2.47% (2/81) Her2-overexpressing breast cancer (**Table 2**).

**Table 1:** Demographic and clinical features of T1 and T2 stage UEBC.

Variables, n(%)	T1 stage UEBC (n=59)	T2 stage UEBC (n=22)	*P* value
Age, yr			0.093
<40	9(15.3)	6(27.3)	
40–50	28(47.5)	13(59.1)	
>50	22(37.3)	3(13.6)	
Body mass index ≥28	2(3.4)	1(4.5)	1.000
Alcohol addiction	1(1.7)	0(0)	1.000
Smoking habit	0	0	─
Family history of breast cancer	0	0	─
History of breast benign lesion biopsy	8(13.6)	2(9.1)	0.720
Age ≤12 yr at menarche	10(16.9)	2(9.1)	0.500
History of abortion	17(28.8)	7(31.8)	0.790
No childbearing/age ≥30 yr at first childbearing	2(3.4)	0(0)	1.000
Duration of hormone replacement therapy >6 mo	2(3.4)	0(0)	1.000
Age ≥55 yr at menopause	4(6.8)	0(0)	0.570
Dense breast parenchyma	45(76.3)	11(50.0)	0.023
Presence of risk factor of breast cancer	57(96.6)	18(81.8)	0.040
History of diabetes	0	0	─
History of high blood pressure	1(1.7)	0(0)	1.000
Multiple lesions in cancerous breast^a^	12(20.3)	5(22.7)	0.770
routine ultrasound screening for breast cancer	26(44.1)	3(13.6)	0.011

a More than two lesions excised. UEBC, unexpected breast cancer.

**Table 2:** Pathological and immunohistological features of T1 and T2 stage UEBC.

Variables, n(%)	T1 stage UEBC (n=59)	T2 stage UEBC (n=22)	*P* value
Pathological type			0.150
Mucinous breast carcinoma	0(0)	1(4.5)	
Invasive ductal carcinoma	57(96.6)	19(86.4)	
Invasive lobular carcinoma	1(1.7)	2(9.1)	
Tubular carcinoma	1(1.7)	0(0)	
**Histological grade** ^a^			0.122
I (well differentiated)	5(8.9)	0(0)	
II (moderately differentiated	43(76.8)	12(66.7)	
III (poorly differentiated	8(14.3)	6(33.3)	
**Molecular subtyping** ^b^			0.304
Luminal A	18(41.9)	3(20.0)	
Luminal B	11(25.6)	7(46.7)	
Her 2-overexpressing	2(4.7)	0(0)	
Triple negative	12(27.9)	5(33.3)	

a 7 of 81 UEBCs did not have histological grade in pathological reports.

b 23 of 81 specimens did not undergo immunohistochemical staining.

UEBC, unexpected breast cancer.

### 3.2 Preoperative US

We retrospectively reviewed the US records and images and found that 59.3% (48/81) were classified into BI-RADS 4a category, 76.5% (62/81) had a circumscribed margin, and 72.8% (59/81) had a regular shape, 95.1% (77/81) presented as hypoechoic masses, 97.5% (79/81) with parallel orientation, 2.5% (2/81) with posterior echo shadowing, and 3.7% (3/81) with microcalcification. All UEBCs were hypovascular(blood flow 0-1 grade). The interobserver agreement varied from substantial for margin and shape to perfect for internal echogenicity, orientation, posterior echo pattern and BI-RADS categorization. The differences in the above lesion descriptors were not significant (**Table 3**).

**Table 3:** Sonographic features of T1 and T2 stage UEBC.

Variables, n (%)	T1 stage UEBC (n=59)	T2 stage UEBC (n=22)	κ coefficient	*P* value
Circumscribed margin	47(79.7)	15(68.2)	0.68	0.278
Regular shape	45(76.3)	14(63.6)	0.78	0.255
Hypoechoic mass	56(94.9)	21(95.5)	0.9	1.000
Parallel orientation	57(96.6)	22(100.0)	0.85	1.000
Posterior echo shadowing	1(1.7)	1(4.5)	0.81	0.472
Internal microcalcification	2(3.4)	1(4.5)	─	1.000
Blood flow 0-1 grade	59(100.0)	22(100.0)	─	─
BI-RADS 4a category	33(55.9)	15(68.2)	0.85	0.318

BI-RADS, Breast Imaging Reporting and Data System; UEBC, unexpected breast cancer.

## 4 Discussion

US-VAEB was used for the first time in 1994, and initially, it was mainly for diagnosis of suspicious breast lesions. In 2004, the US Food and Drug Administration approved US-VAEB for therapeutic excision of breast benign lesions. Currently in China, US-VAEB is widely used for complete excision of BI-RADS grade 3 or 4a lesions that appear for the first time in patients with high-risk factors for breast cancer. BI-RADS grade 4b and 5 lesions are usually recommended for core-needle or fine-needle aspiration biopsy. To date, 19 306 patients with breast abnormalities have undergone this operation in West China Hospital, and only 0.42%(81/19296) were confirmed as breast cancer. The efficiency and safety of US-VAEB for benign breast lesions are considered to be favorable. A meta-analysis (12) of 26 studies involving 18 170 cases showed that the pooled complete resection rate of US-VAEB was 0.930, the recurrence rate was 0.039, and postoperative hematoma, pain and ecchymosis rates after US-VAEB were 0.092, 0.082, and 0.075, respectively. These results indicate that US-VAEB is a reasonable option for low-risk benign lesions for both diagnostic and therapeutic purposes. After US-VAEB, clinical management regulations depend on pathological results. In a clinical practice guideline for US-VAEB (13), expert groups recommend that open surgical excision should be performed when the lesion is confirmed to be completely removed and histopathologically diagnosed as breast cancer, atypical ductal hyperplasia, or borderline or malignant phyllodes tumor, while surveillance is appropriate for benign lesions and others. The most common complications after US-VAEB are hematoma and pain. Small-volume hematoma requires no surgical intervention, and surgery for hemostasis or debridement is necessary only if a patient is suspected of having active bleeding or large hematoma causing severe pain (14).

In the literature, the incidence of UEBC diagnosed by US-VAEB was 1.1–3.4% (15–17). In our study, it was lower at 0.42%. The difference may have resulted from the different inclusion criteria. In some previous studies, cases with BI-RADS 4b or greater lesions that were diagnosed as benign or atypical ductal hyperplasia using core needle or fine needle aspiration biopsy before US-VAEB were included for analysis, while such lesions were excluded in our study.

Of the 81 UEBCs in our study, 22 were T2 stage, with a ratio of 27.16%. No reports concerning T2 stage UEBC were found in the literature. **Table 1** shows that 69.1% (56/81) of UEBCs occurred in patients aged <50 years, which was higher than 66.7% in a previous study by Zheng et al. (17). The difference may have been caused by random error. There was no significant difference in age between T1 and T2 stage UEBC.

The presence of risk factors for breast cancer has a significant influence on clinical management of breast lesions. In general clinical practice, BI-RADS 3 lesions in patients with risk factors for breast cancer are recommended to undergo US-VAEB, while those without risk factors are not. Our study showed that more patients with T1 stage UEBC than T2 stage UEBC had risk factors for breast cancer [96.6% (57/59) vs 81.8% (18/22), *P*=0.040]. Among the 12 types of risk factors for breast cancer in **Table 1**, only breast parenchyma type differed significantly between T1 and T2 stage UEBC (*P*=0.023). Our results indicated that presence of risk factors for breast cancer, especially dense breast parenchyma, correlated with T stage of UEBC. The patients with risk factors for breast cancer were more likely to be diagnosed with breast cancer earlier using US-VAEB. The presence of risk factors motivated patients with breast abnormalities to receive more aggressive treatment such as US-VAEB, rather than routine follow-up. With the widespread popularization of breast cancer prevention and screening, women, especially those with risk factors for breast cancer, are becoming more health conscious and broadly accept US-VAEB as a treatment for benign-looking lesions. Consequently, breast cancer is more likely to be discovered earlier in women with risk factors for breast cancer.

Screening plays an important role in the detection, diagnosis and prognosis of breast cancer. Previous studies have demonstrated that routine screening was able to facilitate early detection of breast cancer and reduce mortality (18–22).In China, the sensitivity of mammography for breast cancer ranged from 47.8% to 64.4% (23) because most women have dense breast paraenchyma. Routine breast screening by US is the predominant method for detection of breast cancer. In our study, the difference in routine breast screening by US was significant between T1 and T2 stage UEBC [44.1%(26/59) vs 13.6%(3/22), p=0.011]. The patients with T1 stage UEBC seemed to prefer routine breast screening by US compared with those with T2 stage UEBC. The absence of routine breast screening was blamed for the late diagnosis of T2 stage UBEC, which emphasized the importance of routine breast screening by US for early detection of breast cancer.

The lesions in our study all were assigned preoperatively to BI-RADS 3 or 4a, and it was possible that they would have been benign before US-VAEB. BI-RADS 4a category is one of the indications for UE-VAEB (13). The interpretation of US images and final assessment of BI-RADS category were subjective; therefore, we invited two experienced radiologists to review all the US images of UEBC. This showed that interobserver agreement varied from substantial for margins and shape to perfect for internal echogenicity, orientation, posterior echo pattern, and BI-RADS category. The description of US images and BI-RADS category were reliable. **Table 3** shows that the distribution of lesion descriptors between T1 and T2 stage UEBC did not differ significantly, which meant that the sonographic features had no correlation with T stage of UEBC, and did not influence early detection of breast cancer.

Our study had several limitations. Firstly, all US images were static and two-dimensional. Secondly, this was a single-center study, with a small sample size and only 22 cases of T2 stage UEBC. A future study with a larger sample size, especially of T2 stage UEBC, is required.

## 5 Conclusion

The occurrence rate of UEBC after US-VAEB was low, and only a small number were at clinical T2 stage. UEBC patients who underwent routine breast screening with US or who presented with risk factors for breast cancer were more likely to be detected at an earlier clinical stage by US-VAEB. It is necessary to perform routine breast screening for early diagnosis of breast cancer.

## Data availability statement

The original contributions presented in the study are included in the article/supplementary material. Further inquiries can be directed to the corresponding author.

## Author contributions

All authors contributed to the study conception and design. Material preparation and drafting of manuscript were performed by WZ. Methodology was determined by HL and HZ. Writing-review and editing were performed by YP. All authors read and approved the final manuscript. All authors contributed to the article and approved the submitted version.
